# Aberrant epigenetic changes and gene expression in cloned cattle dying around birth

**DOI:** 10.1186/1471-213X-8-14

**Published:** 2008-02-11

**Authors:** Li Lin, Qiang Li, Lei Zhang, Dingsheng Zhao, Yunping Dai, Ning Li

**Affiliations:** 1 State Key Laboratory for Agrobiotechnology, China Agricultural University, Yuanmingyuan West Road 2, Beijing 100094, P. R. China; 2 Institute of Space Medico-Engineering, Yuanmingyuan West Road 2, Beijing 100094, P. R. China

## Abstract

**Background:**

Aberrant reprogramming of donor somatic cell nuclei may result in many severe problems in animal cloning. To assess the extent of abnormal epigenetic modifications and gene expression in clones, we simultaneously examined DNA methylation, histone H4 acetylation and expression of six genes (*β-actin*, *VEGF*, *oct4*, *TERT*, *H19 *and *Igf2*) and a repetitive sequence (*art2*) in five organs (heart, liver, spleen, lung and kidney) from two cloned cattle groups that had died at different stages. In the ED group (early death, n = 3), the cloned cattle died in the perinatal period. The cattle in the LD group (late death, n = 3) died after the perinatal period. Normally reproduced cattle served as a control group (n = 3).

**Results:**

Aberrant DNA methylation, histone H4 acetylation and gene expression were observed in both cloned groups. The ED group showed relatively fewer severe DNA methylation abnormalities (p < 0.05) but more abnormal histone H4 acetylations (p < 0.05) and more abnormal expression (p < 0.05) of the selected genes compared to the LD group. However, our data also suggest no widespread gene expression abnormalities in the organs of the dead clones.

**Conclusion:**

Deaths of clones may be ascribed to abnormal expression of a very limited number of genes.

## Background

More and more mammals have been successfully cloned by somatic cell nuclear transfer (SCNT) [1-8]. The new technology attracts great interest because of its potential applications in biomedicine and husbandry [9,10]. However, cloned animals show many abnormalities including low birth rate, placental dysfunctions and large offspring syndrome [11], which collectively appear to be a great barrier to efficient cloning. In SCNT, somatic nuclei must be remodeled from highly differentiated somatic patterns to a totipotent embryonic pattern in order to support early development. This process is called epigenetic reprogramming [12] and is regarded as a reverse of cell differentiation [13]. The reprogramming process is crucial for embryo cloning and determines whether development will continue [14]. It is generally held that incomplete or aberrant reprogramming of donor somatic cell nuclei results in low cloning efficiency and developmental abnormalities.

DNA methylation and histone modification play important roles in genome reprogramming and expression of genes that control animal development. DNA methylation is a major epigenetic modification of the genome and is crucial for genomic stability. It usually occurs at the 5' position of cytosine in symmetrical 5'-CpG-3' dinucleotides. Clusters of CpGs, termed CpG islands [15], are found in the promoter or the first exon of a gene in most cases [16]. Generally, methylation of CpG islands represses the initiation of transcription [17]. Histone amino termini are subject to many dynamic sets of covalent modifications that are thought to be involved in modulating important physiological activities including gene expression [18]. One such modification, histone acetylation, generally takes place in active chromatin regions and is associated with the activation of gene expression. It participates in regulating transcription during the development of preimplantation embryos.

Epigenetic abnormalities in DNA methylation and histone acetylation are common in cloned mammalian embryos or fetuses. A genome-wide reduction in cytosine methylation has been observed in cloned fetuses [19]. Defective demethylation and precocious *de novo *methylation on a genome-wide scale, and aberrant demethylation of some repetitive DNA elements, have been observed in preimplantation embryos [20,21]. Furthermore, DNA methylation of Igf2 DMRs not only vary in different tissues but are also extensively changed during the perinatal period in normal mouse tissues[22]. Higher levels of histone acetylation were observed in swamp buffalo SCNT embryos than in in vitro fertilized (IVF) embryos at the 4- and 8-cell stages [23]. The levels of acetylated histone H4-lysine 5 at the 8-cell stage were lower in cloned than in IVF bovine embryos, and it was suggested that these abnormalities may be caused by the memory of the somatic chromatin pattern [24]. These observations indicate that epigenetic abnormalities occur frequently during the early development of cloned embryos. However, little is known about DNA methylation and histone acetylation in cloned mammals after birth or about the incidence of aberrant epigenetic modifications.

Several genes have been shown to be crucial for embryo development, organogenesis and growth. *β-actin *is a housekeeping gene [25] expressed in most eukaryotic cells and takes part in many physiological processes [25,26]. Vascular endothelial growth factor (*VEGF*) is a pivotal factor promoting the formation of blood vessels in vasculogenesis and angiogenesis. The reduction in *VEGF *expression during embryo development causes decreased angiogenesis and is ultimately lethal [27-29]. *Oct4 *is a mammalian POU transcription factor and is expressed in all totipotent and pluripotent cells [30]. Mammalian telomerase is essential for maintaining telomere length [31], and the *TERT *gene product is part of the catalytic core of this enzyme. Imprinted genes play essential roles in the early development of mammals [32,33]. Maternal expression of the histocompatibility gene (*H19*) and paternal expression of insulin-like growth factor2 (*Igf2*) are observed in cattle [34,35]. *Art2 *is an alu-like repetitive element with at least 100,000 copies in the bovine genome [36].

In the present study, we examined DNA methylation and histone H4 acetylation and expression in the six genes mentioned above, and in a repetitive sequence, in heart, liver, spleen, lung and kidney from cloned cattle that had died at different stages. We aimed to reveal epigenetic and gene expression changes in these clones.

## Results

### DNA methylation of the six genes and the art2 repetitive sequence in five organs from controls and cloned cattle

CpG islands located in the six selected genes and the *art2 *repetitive sequence were predicted using online software and amplified by PCR. The sizes of the amplified fragments and the number of CpG sites in each island are shown in Additional File 2, Table S1. The identities of all amplified PCR products were verified by sequencing. The methylation patterns of the CpG islands were determined using bisulfite-assisted sequencing. Detailed results are shown in Additional File 1: Data of DNA methylation, histone acetylation and gene expression in individual cattle.

Percentage DNA methylation in the six genes and the *art2 *repetitive sequence in heart, liver, spleen, lung and kidney from cloned cattle and controls are shown in Table 1. In the controls (group N), DNA methylation did not vary much among individuals or even among different organs from the same animal, suggesting that DNA methylation is maintained at approximately steady levels in normal cattle.

**Table 1:** DNA methylation in five organs of normal control and dead cloned cattle^a^

	Group	Heart (%)	Liver (%)	Spleen (%)	Lung (%)	Kidney (%)
*β-actin*	N	39.41 ± 1.02	41.37 ± 1.48	39.80 ± 0.90	40.98 ± 0.34	39.02 ± 1.70
	ED	38.63 ± 0.33	39.22 ± 0.90	38.82 ± 1.18	40.39 ± 0.34	39.81 ± 1.36
	LD	38.43 ± 0.90	39.61 ± 0.34	39.80 ± 0.90	39.61 ± 0.68^b^	39.80 ± 0.34
*VEGF*	N	6.81 ± 2.55	2.46 ± 0.66	2.75 ± 1.09	6.67 ± 4.40	7.54 ± 2.65
	ED	8.40 ± 6.19	2.46 ± 0.91	4.53 ± 2.34	5.36 ± 2.40	3.48 ± 3.28
	LD	7.68 ± 3.48	7.10 ± 4.57	6.39 ± 4.39^b^	4.20 ± 0.66	8.99 ± 3.38
*art2*	N	30.00 ± 3.46	36.00 ± 2.00	40.67 ± 4.62	40.67 ± 4.16	38.00 ± 10.58
	ED	35.33 ± 9.24	30.00 ± 2.00	35.33 ± 11.55	38.67 ± 13.32	38.00 ± 8.72
	LD	39.33 ± 4.16	31.33 ± 5.77	35.33 ± 18.90	29.33 ± 12.06	39.33 ± 3.06
*oct4*	N	59.36 ± 10.90	58.64 ± 10.43	53.75 ± 8.55	51.30 ± 6.52	44.97 ± 10.68
	ED	40.56 ± 10.21	64.55 ± 2.27	59.86 ± 5.22	74.31 ± 5.88^b^	37.94 ± 16.72
	LD	40.44 ± 6.14	54.47 ± 3.67	49.08 ± 6.12	66.75 ± 8.95^b^	38.97 ± 5.68
*TERT*	N	70.83 ± 4.39	65.55 ± 7.09	72.50 ± 13.23	70.70 ± 13.18	75.97 ± 8.75
	ED	73.61 ± 14.00	73.33 ± 2.92	78.33 ± 6.86	75.56 ± 6.25	77.50 ± 9.46
	LD	74.44 ± 11.13	81.67 ± 3.31^b^	82.08 ± 2.20	87.08 ± 4.81	80.50 ± 11.82
*H19*	N	75.00 ± 18.33	71.11 ± 5.14	63.75 ± 20.31	85.28 ± 7.13	62.64 ± 7.34
	ED	83.89 ± 12.46	64.31 ± 10.48	72.36 ± 3.39	76.11 ± 12.25	57.36 ± 15.73
	LD	87.36 ± 7.89	64.17 ± 10.10	74.72 ± 9.22^b^	83.34 ± 12.77	65.97 ± 10.97
*Igf2*	N	85.56 ± 2.68	75.83 ± 18.39	85.00 ± 7.64	81.39 ± 12.62	74.17 ± 2.2
	ED	83.61 ± 5.43	65.83 ± 14.53	91.39 ± 3.37	86.95 ± 8.91	63.33 ± 6.29^b^
	LD	95.83 ± 0.84^b^	83.05 ± 2.92	90.00 ± 3.64	88.61 ± 2.93	83.89 ± 2.93^b^

^a ^Values are presented as the mean ± SEM. N denotes normal control, ED denotes early death group, and LD denotes late death group.

^b ^denotes significant difference (p < 0.05) between ED and N, LD and N.

Aberrant DNA methylation was found in the six selected genes but not in *art2 *in the different organs of the ED and LD groups compared with the controls. Detailed results are shown in Table 2. In the ED group, methylation of *oct4 *was elevated in lung, while methylation of *Igf2 *was reduced in kidney. In the LD group, *β-actin *methylation was decreased in lung. In contrast, there were higher levels of methylation of *VEGF *in spleen, *oct4 *in lung, *TERT *in liver, *H19 *in spleen and *Igf2 *in heart and kidney (*P *< 0.05). Apparently there was more aberrant DNA methylation in the LD group than the ED group.

**Table 2:** Variations of DNA methylation in two cloned groups compared to normal control^a^

	Group	Heart	Liver	Spleen	Lung	Kidney
*β-actin*	ED					
	LD				Down	
*VEGF*	ED					
	LD			Up		
*art2*	ED					
	LD					
*Oct4*	ED				Up	
	LD				Up	
*TERT*	ED					
	LD		Up			
*H19*	ED					
	LD			Up		
*Igf2*	ED					Down
	LD	Up				Up

^a ^Up, denote a significantly higher of DNA methylation than normal control. Down, denote a significantly lower of DNA methylation than normal control. ED denotes early death group and LD denotes late death group.

### Histone H4 acetylation in the six selected genes and the art2 repetitive sequence in five organs from the cloned cattle

Aberrant histone H4 acetylation was found in organs from both two cloned groups. Detailed results are shown in Table 3. All six genes and the *art2 *repetitive sequence in the ED group and four genes in the LD group, showed aberrant histone H4 acetylation in different organs. Acetylation of *art2 *was shown to be aberrant in all five organs from the ED group, but only in liver and lung from the LD group. Aberrant acetylation of *β-actin *was observed in lung and kidney from the ED group and in spleen and kidney from the LD group. *VEGF *showed aberrant acetylation in heart and lung from the ED group and in liver, lung and kidney from the LD group. *oct4 *showed aberrant acetylation in liver, spleen and lung from the ED group and in heart and lung from the LD group. *TERT *showed decreased acetylation in kidney from the ED group. Two imprinted genes, *H19 *and *Igf2*, showed a tendency towards increased acetylation in liver, spleen, heart, lung and kidney of the ED group.

**Table 3:** Relative histone H4 acetylation in five organs of dead cloned cattle compared to normal control^a^

	Group	Heart	Liver	Spleen	Lung	Kidney
*β-actin*	N	1.00 ± 0.30	1.00 ± 0.17	1.00 ± 0.18	1.00 ± 0.12	1.00 ± 0.08
	ED	0.82 ± 0.16	0.81 ± 0.32	1.02 ± 0.22	0.47 ± 0.15^b^	2.33 ± 0.13^c^
	LD	1.26 ± 0.17	0.63 ± 0.15	0.61 ± 0.12^b^	0.82 ± 0.18	3.12 ± 0.79^c^
*VEGF*	N	1.00 ± 0.51	1.00 ± 0.31	1.00 ± 0.38	1.00 ± 0.23	1.00 ± 0.53
	N	2.14 ± 0.09^b^	0.76 ± 0.19	1.07 ± 0.18	0.60 ± 0.04^b^	1.93 ± 0.59
	LD	1.23 ± 0.16	0.47 ± 0.06^b^	1.27 ± 0.26	0.66 ± 0.08^b^	3.23 ± 0.49^c^
*art2*	N	1.00 ± 0.20	1.00 ± 0.54	1.00 ± 0.64	1.00 ± 0.40	1.00 ± 0.31
	ED	0.49 ± 0.01^c^	5.54 ± 2.10^b^	2.88 ± 0.70^c^	4.49 ± 0.94^c^	0.47 ± 0.09^b^
	LD	0.92 ± 0.12	3.53 ± 0.89^b^	0.35 ± 0.17	6.36 ± 1.03^c^	0.70 ± 0.11
*oct4*	N	1.00 ± 0.33	1.00 ± 0.60	1.00 ± 0.31	1.00 ± 0.44	1.00 ± 0.46
	ED	1.36 ± 0.96	4.61 ± 0.71^c^	1.96 ± 0.52^b^	0.23 ± 0.09^b^	0.58 ± 0.19
	LD	3.33 ± 0.40^c^	1.52 ± 0.14	1.12 ± 0.05	0.37 ± 0.17^b^	0.47 ± 0.27
*TERT*	N	1.00 ± 0.62	1.00 ± 0.68	1.00 ± 0.35	1.00 ± 0.37	1.00 ± 0.40
	ED	1.70 ± 0.49	0.88 ± 0.32	1.74 ± 0.59	0.75 ± 0.19	0.51 ± 0.10^b^
	LD	0.55 ± 0.27	0.63 ± 0.10	0.44 ± 0.02	0.81 ± 0.14	0.91 ± 0.15
*H19*	N	1.00 ± 0.37	1.00 ± 0.40	1.00 ± 0.19	1.00 ± 0.27	1.00 ± 0.49
	ED	1.22 ± 0.40	1.78 ± 0.40^b^	2.92 ± 0.24^c^	0.73 ± 0.21	0.96 ± 0.40
	LD	0.79 ± 0.16	0.56 ± 0.12	0.62 ± 0.18	0.62 ± 0.17	2.02 ± 1.01
*Igf2*	N	1.00 ± 0.08	1.00 ± 0.04	1.00 ± 0.62	1.00 ± 0.30	1.00 ± 0.27
	ED	2.58 ± 0.84^b^	0.90 ± 0.20	1.16 ± 0.43	2.10 ± 0.47^b^	2.26 ± 0.80^b^
	LD	1.07 ± 0.10	0.72 ± 0.21	0.37 ± 0.03	0.79 ± 0.05	1.37 ± 0.34

^a ^The relative histone H4 acetylation in two groups compared with normal control (the relative hiotone H4 acetylation of normal control were normalized to 1). Values are presented as the mean ± SEM. N denotes normal control, ED denotes early death group and LD denotes late death group.

^b ^(p < 0.05), ^c ^(p < 0.01) denote significant differences between ED and N, LD and N.

As summarized in Table 4, abnormalities in histone H4 acetylation were observed in heart, liver, spleen, lung and kidney from both cloned groups. The ED group showed more abnormal histone H4 acetylation than the LD group. In heart, ED showed three aberrantly acetylatedes genes and LD showed one. In liver, spleen, lung and kidney, ED showed 3, 3, 5 and 4 aberrantly acetylatedes genes respectively, and LD showed 2, 1, 3 and 2 respectively. In the ED group, the repetitive sequence *art2 *showed aberrant acetylation in all five organs, but in the LD group it was aberrantly acetylated only in liver and lung. In the ED group, *β-actin, VEGF, oct4, TERT, H19 *and *Igf2 *showed aberrant acetylation in 2, 2, 3, 1, 2 and 3 organs respectively, while in the LD group they were aberrantly acetylated in 2, 3, 2, 0, 0 and 0 organs respectively. These data suggest that histone H4 acetylation was more frequently abnormal in the ED group, which also showed more pathological organs (Table 4).

**Table 4:** Principal abnormalities at necropsy of dead cloned cattle^a^

**Cattle**	**Heart**	**Liver**	**Spleen**	**Lung**	**Kidney**
**ED1**	Hypertrophy, patent foramen ovale	Hepatomegaly, congestion	Normal	Atelectasis, thickened alveolar wall	Normal
**ED2**	Valvular agenesis	Hepatomegaly	Hemorrhage	Atelectasis, thickened alveolar wall	Normal
**ED3**	Hypertrophy, hemorrhage, necrosis, thickened cardiac muscle, patent foramen ovale	Hepatomegaly, congestion	Hemorrhage, two lobes	Congestion, atelectasis, disjunct six lung lobes	Normal
**LD1**	Volvulus induced death, no other obvious organic pathological changes				
**LD2**	Arthritis induces paralysis, no other obvious organic pathological changes				
**LD3**	Intestinal spasm, no other obvious organic pathological changes				

^a ^All samples were diagnosed by a qualified veterinarian

**Table 5:** Variations of histone H4 acetylation in two groups of cloned cattle compared to normal control^a^

	Group	Heart	Liver	Spleen	Lung	Kidney	Organs affected
*β-actin*	ED				Down	Up	2
	LD			Down		Up	2
*VEGF*	ED	Up			Down		2
	LD		Down		Down	Up	3
*art2*	ED	Down	Up	Up	Up	Down	5
	LD		Up		Up		2
*Oct4*	ED		Up	Up	Down		3
	LD	Up			Down		2
*TERT*	ED					Down	1
	LD						0
*H19*	ED		Up	Up			2
	LD						0
*Igf2*	ED	Up			Up	Up	3
	LD						0
*Abettant genes*	ED	3	3	3	5	4	18^b^
	LD	1	2	1	3	2	9^b^

^a ^Up, denote a significantly higher histone H4 acetylation than normal control. Down, denote a significantly lower histone H4 acetylation than normal control. ED denotes early death group and LD denotes late death group.

^b ^Total

### Expression of β-actin, VEGF, H19 and Igf2

*Oct4 *is only expressed in the germ cells and during early embryo development [30], and *TERT *is not expressed in somatic cells [37]. We therefore only checked the expression of *β-actin*, *VEGF*, *H19 *and *Igf2 *in the five tissues (heart, liver, spleen, lung and kidney) from normal and cloned cattle by real-time quantitative RT-PCR. The amplified products were identified by melting curve profile analysis and sequencing. The final data showed the relative transcript abundance of each target gene normalized to *GAPDH*.

The relative expression levels of the 4 genes in 5 organs from control and cloned cattle are shown in Table 6. Statistically significant differences between cloned cattle and controls were found in gene expression levels in several organs (Table 7). In the ED group, *β-actin *expression was increased in heart and reduced in spleen compared with controls. For *VEGF, H19 *and *Igf2*, there was a tendency towards greater expression in the different organs from the cloned cattle. *VEGF *expression was elevated in spleen and kidney, while *H19 *(*P *< 0.01) and *Igf2 *expression were elevated in heart. In the LD group, only *Igf2 *expression was reduced in lung (*P *< 0.05). Most aberrant gene expression was found in the ED group.

**Table 6:** Expression of four genes in five organs of dead cloned cattle compared to normal control^a^

*Gene*	Organ Group	Heart	Liver	Spleen	Lung	Kidney
*β-actin*	N	1.00 ± 0.37	1.00 ± 0.50	1.00 ± 0.60	1.00 ± 1.08	1.00 ± 0.72
	ED	4.44 ± 0.40^b^	0.89 ± 0.32	0.20 ± 0.03^b^	0.21 ± 0.01	0.41 ± 0.10
	LD	2.57 ± 1.61	0.45 ± 0.36	0.45 ± 0.27	2.05 ± 2.57	0.34 ± 0.15
*VEGF*	N	1.00 ± 0.42	1.00 ± 0.91	1.00 ± 0.44	1.00 ± 0.39	1.00 ± 0.79
	ED	3.62 ± 2.33	2.92 ± 1.33	1.89 ± 0.04^b^	0.96 ± 0.20	4.55 ± 1.37^b^
	LD	1.90 ± 1.14	1.13 ± 0.32	0.49 ± 0.21	0.63 ± 0.25	2.35 ± 0.69
*H19*	N	1.00 ± 0.48	1.00 ± 0.35	1.00 ± 0.31	1.00 ± 1.13	1.00 ± 0.72
	ED	10.00 ± 3.67^c^	26.38 ± 30.17	1.51 ± 1.08	0.54 ± 0.26	1.83 ± 1.51
	LD	0.71 ± 0.16	0.08 ± 0.04	0.17 ± 0.14	0.53 ± 0.71	1.91 ± 0.91
*Igf2*	N	1.00 ± 0.25	1.00 ± 0.09	1.00 ± 0.60	1.00 ± 0.51	1.00 ± 0.93
	ED	18.97 ± 12.83^b^	9.85 ± 11.83	1.49 ± 0.42	1.02 ± 0.28	2.18 ± 2.54
	LD	1.15 ± 0.21	1.05 ± 0.77	0.40 ± 0.59	0.21 ± 0.12^b^	0.91 ± 0.37

^a ^The relative trancripts in two groups compared with normal control (the relative trancripts of normal control were normalized to 1). Values are presented as the mean ± SEM. N denotes normal control, ED denotes early death group and LD denotes late death group.

^b ^(p < 0.05), ^c ^(p < 0.01) denote significant differences between ED and N, LD and N.

**Table 7:** Variations of expression in two groups of cloned cattle compared to normal control^a^

Gene	Group	Heart	Liver	Spleen	Lung	Kidney	Organs affected
*β-actin*	ED	Up		Down			2
	LD						0
*VEGF*	ED			Up		Up	2
	LD						0
*H19*	ED	Up					1
	LD						0
*Igf2*	ED	Up					1
	LD				Down		1
*Aberrant genes*	ED	3	0	2	0	1	6^b^
	LD	0	0	0	1	0	1^b^

^a ^Up, denote a significantly higher gene rexpression than normal control. Down, denote a significantly lower gene rexpression than normal control. ED denotes early death group and LD denotes late death group.

^b ^Total

### Overall, DNA methylation, histone H4 acetylation and gene expression tended to be elevated in the cloned cattle

Putting all the cloned cattle samples together, we found that DNA methylation, histone H4 acetylation and gene expression tended to be higher than in controls. The ratio of up-:down-regulation was 7:2 for DNA methylation, 17:10 for histone H4 acetylation and 5:2 for gene expression.

## Discussion

Many studies have suggested that incomplete or aberrant reprogramming of donor somatic cell nuclei, including abnormal DNA methylation and histone acetylation, causes abnormal development of animal clones [20,21,23,24]. Previous investigations of epigenetic reprogramming have mostly been performed on the genome scale. To date, changes in the epigenetic status of specific genes in embryos and organs of natal clones remain elusive. To our knowledge, this is the first time that DNA methylation, histone H4 acetylation and gene expression have been examined simultaneously for specific genes in cloned cattle.

We found aberrant DNA methylation, histone H4 acetylation and gene expression in different organs from two groups of cloned cattle. The ED group showed fewer severe DNA methylation abnormalities than the LD group, though the abnormalities in histone H4 acetylation and gene expression were more marked. Overall, DNA methylation, histone H4 acetylation and gene expression tended to be greater in cloned cattle than in controls. Our data also suggest that there are no widespread gene expression abnormalities in organs from clones that have died, so the death of clones may be ascribed to abnormal expression of a very limited number of pivotal genes.

Abnormal expression of a few key genes suffices to cause severe consequences. Carmeliet and Ferrara found that lack of a single VEGF allele was lethal in embryos [27,28]. Disruption of imprinted genes (biallelic expression) resulted in overgrowth of the fetus and placenta in mice [38] and severe congenital disorders in humans such as Beckwith-Wiedemann syndrome (BWS), Prader-Willi syndrome (PWS) and Angelman syndrome (AS) [39]. As aberrant expression of a few genes could cause the death of cloned cattle, these causal gene(s) may be different in different cases. Many studies have revealed aberrant expression of genes in cloned animals [40-47], including imprinted genes [43], X-linked genes [48], apoptosis-related genes [42] and other development-related genes. Our data also reveal aberrant expression of imprinted genes and development-related genes in a few organs. Although many gene expression abnormalities have been reported in clones, it is difficult to know to what extent these abnormalities could induce clone death.

DNA methylation is important in regulating gene expression [49]. Appropriate DNA methylation is essential for normal gene function [50]. Recent studies have revealed aberrant methylation of repetitive elements and imprinted genes in cloned embryos during preimplantation development [21,51]. In the present study, we found more severely aberrant DNA methylation in the LD group than the ED group. It is well known that the mammalian genome undergoes two major rounds of epigenetic reprogramming: during germline development and during preimplantation development [52-54]. Appropriate reprogramming of DNA methylation in the early embryo is crucial for the subsequent development of cloned animals. We speculate that relatively normal reprogramming of methylation occurred during the early development of our cloned cattle, otherwise these animals would not have developed to term. On the other hand, it is reported that epigenetic status can be influenced by environmental factors [55]. As our results indicate that DNA methylation was more variable in cloned cattle that died after the perinatal period than in those that died during that period, we speculate that variations in DNA methylation after birth are sensitive to environmental factors.

More aberrant histone H4 acetylation changes were found in the ED group than in the LD group. These abnormalities of acetylation probably occurred randomly during the development of the cloned cattle. Overall, however, there was a tendency towards greater histone H4 acetylation in the cloned cattle. In particular, the imprinted genes, *H19 *and *Igf2*, both showed elevated acetylation levels in all aberrant organs. The increased acetylation levels are noteworthy because crucial aspects of mammalian physiological activity such as reproduction, placentation and energy homeostasis are regulated by imprinted genes [56]. It is reasonable to speculate that if elevated H4 acetylation causes dramatic changes of expression of key gene(s), the animal will die or continue to develop abnormally.

More abnormal acetylation and variations in gene expression were observed in the ED group, which had more organ pathologies, suggesting a correlation between clone death and aberrant gene expression. A previous study showed that aberrant global histone acetylation of somatic chromatin began at the one-cell stage in SCNT [24]. Cloned embryos are more highly acetylated than controls in the trophectoderm at the blastocyst stage [57]. In dead cloned cattle, aberrant gene expression was found in many types of organs [58]. On the basis of these studies and our own observations, we conclude that histone acetylation and gene expression are reprogrammed randomly throughout early development in cloned animals. Some incorrect reprogramming may be corrected during the development of preimplantation embryos, but uncorrected abnormal histone acetylation and gene expression will persist and may result in severe outcomes such as pathological organ changes in the clones. This speculation is consistent with the opinion that small reprogramming errors in redifferentiation can be magnified during the subsequent development of cloned embryos [59].

It is generally known that gene expression is controlled by multiple factors [60,61]. DNA methylation and histone H4 acetylation are only two of these factors. Others such as histone H3 acetylation and histone methylation are also involved. These histone modifications interact cooperatively to regulate gene expression [62]. Transcription factors also participate in the regulation of gene expression. Our results suggest that histone H4 acetylation and DNA methylation may cooperate with other factors to regulate gene expression. Interactions among these factors may counteract the negative effect of one or more of them. In addition, there is a noticeable tendency for DNA methylation, histone H4 acetylation and gene expression to be elevated, and more data are required to reveal the exact influence of this tendency.

The extent of DNA methylation and histone H4 acetylation in the genome as a whole, and the status of other histone modifications, are still unknown in newborn normal and cloned cattle. Therefore, additional studies are needed to elucidate the significance of these unknown epigenetic variations in the development of normal and cloned cattle.

## Conclusion

Abnormal DNA methylation, histone H4 acetylation and gene expression were found in five organs from both ED and LD cloned cattle groups in this research. However, our study showed that no generalized gene expression abnormalities detected in the dead clones. So, we postulate that the deaths of clones may be due to aberrant expression of a very limited number of genes.

## Methods

### Nuclear transfer and tissue collection

The nuclear transfer procedure and tissue collection have been described elsewhere [58,63]. The donor nuclei were obtained from skin fibroblast cells of Holstein cows (4 years old). The principal abnormalities of the cloned cattle at necropsy were diagnosed by a qualified veterinarian and are shown in Table 1.

### Animals

Three groups of cattle, N, ED and LD, were used in this study. Group N stands for normal controls. This group comprised N1, N2 and N3, three cattle produced by normal sexual reproduction and slaughtered within two days after birth. ED group comprised ED1, ED2 and ED3, which died during the perinatal period and showed many organ pathologies. The LD group comprised LD1, LD2 and LD3, which died at least 6 months after the perinatal period and showed no pathological changes.

### Preparation of DNA and RNA

Genomic DNA was isolated from organs by phenol-chloroform extraction and ethanol precipitation. Total RNA was extracted using a TRIZOL RNA isolation kit (Invitrogen, Carlsbad, CA, USA). The RNA preparations were treated with RNase-free DNase I to eliminate possible genomic DNA contamination and stored at -70°C.

### Bisulfite-assisted genomic sequencing of CpG islands

DNA methylation was analyzed using DNA embedded in low melting point (LMP) agarose and the bisulfite-assisted genomic sequencing method according to Perta Hajkova et al. [64]. A GELaseTM Agarose Gel-Digesting Preparation (Epicentre, Madison, Wisconsin, USA) was used to digest the LMP agarose before PCR.

CpG islands were predicted using online software [65,66]. We designed primers flanking the CpG islands using oligo 6.0 software according to the guidelines for primer design by Perta Hajkova et al. [64]. For *β-actin*, *VEGF, TERT, H19 *and *Igf2*, semi-nested primers were designed for efficient amplification. A gradient PCR cycler (Eppendorf, Hamburg, Germany) was used to optimize the annealing temperature. The sequences of the PCR primers used for amplifying the targeted products are shown in Table S1 of Additional File 2: Primers and PCR parameters.

PCR was performed using 50 ng bisulfite-treated DNA in a final volume of 20 microliters. The PCR began with a denaturing step at 94°C for 5 min followed by 35 cycles of 94°C for 20 s, annealing temperature for 30 s and 72°C for 30 s, and a final elongation step at 72°C for 10 min, in a Perkin-Elmer 9600 or 9700 (Applied Biosystems, Foster City, CA, USA). When semi-nested primers were used, the first-round PCR products were diluted tenfold and used as templates for the second-round PCR. The PCR products were purified using agarose gel elution and cloned into a pMD18-T vector (TaKaRa, Dalian, China). A colony PCR protocol was used to verify the presence of the correct insert. The plasmids with correct insert sizes were sequenced.

### Chromatin immunoprecipitation (CHIP)

Chromatin was immunoprecipitated as described previously [67] with some modifications. Briefly, the tissue samples were homogenized (Qiagen, Hilden, Germany) in 1 ml 1% formaldehyde at 30 Hz for 5 min, then cross-linked for 10 min and washed twice with 1 ml phosphate-buffered saline (PBS). They were then lysed using 1 ml of cell lysis buffer and 1 ml of nuclear lysis buffer successively. Chromatin in the lysis suspension was sheared using a sonicator (Sonics and Materials Inc., Newtown, CT, USA) for 5 rounds of 15 10 s pulses at output level 4 on ice. The sheared suspension was cleared by centrifugation (4°C, 10 min at 12,000 rpm, Eppendorf 5417R, Hamburg, Germany) and was used immediately or stored at -70°C. In our study, 20 μl aliquots of the supernatants were used to extract input DNA and 100 μl aliquots were used in each CHIP reaction. Commercial Salmon Sperm DNA/Protein A Agarose (Upstate, NY, USA) was used as a substitute for the blocked Staph A cells. In addition, 1.5 μg of antibodies against acetylated histone H4 (in general, not against specific residues) (Upstate, 06-866, NY, USA) were used for each sample. The reaction was incubated on a rotating platform at 4°C overnight. Subsequently, 40 μl of Salmon Sperm DNA/Protein A Agarose were added to each sample followed by incubation for 30 min and centrifuging. The pellets were washed twice with 1 × dialysis buffer and four times with IP buffer. IP elution buffer (150 μl) was added and shaken for 15 min on a vortexer (QL901, HaiMen, China); this step was repeated once and the supernatant was collected after centrifugation. RNase A (1 μl of 10 mg/ml) was added, followed by 5 M NaCl to a final concentration of 0.3 M, and the samples were incubated in a 65°C water bath for 4 h to reverse the formaldehyde crosslinks. Then 50 μl of 5× PK buffer and 2.5 μl of proteinase K (10 μg/μl) were added to each sample. After the proteinase K digestion, 170 μl of protein precipitation solution (Promega, Madison, WI, USA) were added, then the mixture was kept on ice for 10 min and centrifuged at 12,000 rpm for 5 min. Finally, CHIP DNA was separated from the supernatant liquid using a DNA purification kit (Promega, Madison, WI, USA) and was eluted in 50 μl water.

### Relative quantification (ΔΔCt) of CHIP DNA

Relative quantification of CHIP DNA was conducted using a 7900 HT system (Applied Biosystems, Foster City, CA, USA) and a SYBR Green qPCR kit. Primers were designed using Primer Express software (Applied Biosystems, Foster City, CA, USA) and are shown in Table S2 of Additional File 2: Primers and PCR parameters.

In the relative quantification (ΔΔCt) of CHIP DNA, 'β-actin' of the CHIP input DNA was used as a positive control. Negative controls were set according to the CHIP protocol. The cycling parameters for PCR were as follows: 95°C for 10 min (1 cycle), then 95°C for 30 s, 60°C (or 56°C and 62°C, varied according to different primers) for 30 s and 72°C for 30 s (40 cycles). The specificity of the amplified products was examined on 2.5% agarose gels.

The quantity of each tested gene compared to controls in organs from different cloned individuals in the ED and LD groups was calculated by formula R = 2^-ΔΔCt^. *β-actin *of the CHIP input DNA was selected as the control gene. The differences between the mean Ct values of the tested genes in CHIP DNA and the positive control gene were denoted ΔCt. The difference between the ΔCt in cloned cattle (ED and LD) and controls was labeled ΔΔCt.

### Reverse transcription and relative quantification (standard curve) of gene transcripts

Reverse transcription reactions were carried out using an RT kit (Invitrogen, Carlsbad, USA) with approximately 1 μg RNA in a total volume of 20 μl and oligo-dT primers.

The expression levels of the four genes were quantified by quantitative real-time PCR on the 7900 HT system using SYBR Green qPCR kit. The primers for quantitative PCR are shown in Table S3 of Additional File 2: Primers and PCR parameters. The PCR reaction mixture (15 μl) contained 7.5 μl SYBR Green qPCR mix, 1 μl forward primer (1.5 μM), 1 μl reverse primer (1.5 μM), 1 μl cDNA template and 4.5 μl H_2_O. PCR was conducted at 95°C (10 min) for initial denaturation, followed by 40 cycles of 15 s at 95°C for DNA denaturation and 1 min at 60°C for primer annealing and extension. The melting protocol was 4 cycles of 15 s at 95°C, 15 s at 60°C and 15 s at 95°C.

Gene expression was quantified using the relative standard curve method. The housekeeping gene *Glyceraldehyde-3-phosphate (GAPDH) *was used as an endogenous RNA control. The amplified *GAPDH *PCR fragment was cloned into a plasmid. Serial dilutions (10–10^6 ^copies/well) of the plasmid were used to form a standard curve of the cycle threshold (Ct) value. The amplification of target genes and *GAPDH *was examined using the same cDNA from one RT-PCR tube for each sample. The amount of mRNA of each gene was determined from the relative standard curve and divided by the mean quantity of *GAPDH *to obtain a relative transcript value. Then the mean and standard deviation of replicates for each sample were calculated.

### Statistical analysis

Data are presented as mean ± SEM. Statistically significant differences among the 3 groups were tested by one-way ANOVA using SPSS software (version 10.0; SPSS, Inc., Chicago, IL, USA). Differences were considered to be statistically significant at the 95% confidence level (*p < 0.05*).

## Authors' contributions

LL carried out the DNA methylation and gene expression studies, participated in data statistical analysis and drafted the manuscript. QL carried out the histone H4 acetylation studies, participated in data statistical analysis and drafted the manuscript. LZ carried out the sample collection. DZ carried out the sequence cloning of *TERT*. YD participated in somatic cell nuclear transfer. NL conceived of the study, and participated in its design and coordination and helped to draft the manuscript. All authors read and approved the final manuscript.

## Supplementary Material

Additional file 1 Data of DNA methylation, histone acetylation and gene expression in individual cattle. The data provided represent the DNA methylation, histone acetylation and gene expression in individual cattle. Click here for file

Additional file 2 Primers and PCR parameters. The table provided shows primers and PCR parameters used for CHIP DNA and mRNA detection. Click here for file
