# Supplementary material for: Aberrant epigenetic changes and gene expression in cloned cattle dying around birth
Source: BMC Dev Biol. 2008 Feb 11;8:14. doi: 10.1186/1471-213X-8-14 (PMC2268668; doi:10.1186/1471-213X-8-14)
Supplement: Additional file 2 — Primers and PCR parameters. The table provided shows primers and PCR parameters used for CHIP DNA and mRNA detection. [file 1471-213X-8-14-S2.pdf]

Table S1. Primer and PCR parameter used for DNA methylation examination[a]

	Primer sequence (5′—3′)	Sequence accession	Location of primer	Position of CpG island	Fragment size(bp)	No. of CpG sites in CpG island	Annealing temperature (℃)
β-actin	F: GTTTTTTTTTTTGGGTATGGAAT<br>F′:TTTTTAATTTTATTATGAAGTGTGA<br>R: CCACATCTACTAAAAAATAAACA	AY141970	828-850<br>872-896<br>1085-1107	Exon	236	17	50<br>50
VEGF	F: TGAGGTAGTTGTGTGTATAGATAGT<br>R′:AAAAAAACAAAAAATTCATAATTTC<br>R: AACCTAAAACCACTTAACATAATAA	NM_174216	368-392<br>540-564<br>599-623	Exon	197	23	50<br>50.7
art2[19])	F: TTTATGTGAAGAGTTGATTTATTGGA<br>R: CCAAACCTCCCTATCCATCTC	X05087	63-83<br>199-224		162	5	51.4
oct4	F: GATTTGGATGAGTTTTTAAGGGTTTT<br>R: ACTCCAACTTCTCCTTATCCAACT	AF022986	3054-3079<br>3323-3346	Exon1	293	24 or 20 [b]	51.6
TERT	F: ATTAATAGAGAATTGGTTTGAGG<br>F′: AGTTTGGAGTTAAGGAAATTAAGT<br>R: AATCCAAAAAAAACTCATAACAAC	DQ399842	302-324<br>374-397<br>632-655	Promoter	282	24	50<br>50.7
H19	F : TAATTTATTTAGTAGTAGGTTATAG<br>R′: CAATATCCCCAAATACACTAAAA<br>R: CCCCTAAATACTATTCTATCTCT	AY849926	582-606<br>831-853<br>895-917	Exon1	272	24	50<br>50.7
Igf2	F: GTTTTGTTTATTTAGGATTTTGTTA<br>F′:GATTAGAAGTGAGTTAAAGTGT<br>R: AATATTATTCTAATCCCCTCAAC	NM_174087	586—610<br>643—664<br>837—859	Exon10 (DMR)	217	12	50<br>55.4

[a] F, forward outer primer; F′, forward inner primer; R, reverse outer primer; R′, reverse inner primer.

[b] The amplified CpG island included 24 CpG sites or 20 CpG sites (an absent 13bp from 243 to 255 nt in some clones).

Table S2. Primer and PCR parameter used for histone H4 acetylation examination [a]

	Primer sequence (5′—3′)	Sequence accession	Position	Fragment size(bp)	Annealing temperature (℃)
β-actin	F: GCTCGCCATCAGTTACAAG<br>R: GGACACACAGACCCGAGAGT	AY293622	420<br>631	231	56
VEGF	F: GAGACCCTGGTGGACATCTTCC<br>R: GCACACACTCCAGACTTTCGTC	NM_174216	708<br>807	121	62
art2	F:CATCCAGCCATCTCATCCTC<br>R:GAGATCCAACCAGTCCATTC	X05087	138<br>316	179	60
Oct4	F:GAGGAAGAGACAAGTGCAAGCAAT<br>R:AGCGTCTAGAGCTACTTGGTCAGC	AF022986	1495<br>1597	103	60
TERT	F:GTTTCCTTCCAACTGTAGCCTCG<br>R:AGCAGTGAGAGCAACGTCTACCTT	DQ399842	3957<br>4058	102	60
H19	F:GGATATCGACGTTCCTTTAGACTC<br>R:AAATGCAGCATATTCTTGAGTCAG	AY849926	1455<br>1702	248	60
Igf2	F: TGCTCCTCTTGTCACTAACACACA<br>R: GCATGGTGACAAATAACATCATACC	NW_930796	360<br>457	98	60

[a] F, forward outer primer; R, reverse outer primer.

Table S3. Primer and PCR parameter used for real-time quantitative PCR [a]

Gene	Primer sequence (5′—3′)	Sequence accession	Position	Fragment size(bp)	Annealing temperature (℃)
GAPDH	F: CGACTTCAACAGCGACACTCAC<br>R: CCCTGTTGCTGTAGCCAAATTC	NM_001034034	918-939<br>1015-1036	118	60
β-actin	F: TGGCGCTTGACTCAGGATTT<br>R: GTACAATCAAGTCCTCGGCCAC	NM_173979	1344-1363<br>1427-1448	105	60
VEGF	F: GAGCGGAGAAAGCATTTGTTTG<br>R: CGTTCGTTTAACTCAAGCTGCC	NM_174216	984-1005<br>1066-1087	104	60
H19	F: AGAGATGGTGCTACCCAGCTCA<br>R: TGTAGTGGTTCCAAAATGCAGC	AY849926	1615-1636<br>1694-1715	101	60
Igf2	F: GCATCGTGGAAGAGTGTTGCTT<br>R: TCGTAGAGGCAGACACATCCCT	NM_174087	302-323<br>382-403	102	60

[a] F, forward outer primer; R, reverse outer primer.
